# A deeply divergent TcdB-like toxin in a cryptic clade C-III *Clostridioides* strain from a One Health genomic survey in Japan

**DOI:** 10.1128/aem.01139-26

**Published:** 2026-09-25

**Authors:** César Rodríguez

**Affiliations:** 1 Facultad de Microbiología & CIET, Universidad de Costa Rica 495716, San José, Costa Rica; Centers for Disease Control and Prevention, Atlanta, Georgia, USA

## Abstract

In a recent One Health genomic survey of community-associated *Clostridioides difficile* in Japan, S. Yoshizawa, K. Komori, K. Aoki, T. Sawa, et al. (Appl Environ Microbiol 92:e00397-26, 2026, https://doi.org/10.1128/aem.00397-26) deposited the genome of strain TUM20227, a human *tcdA*^−^/*tcdB*^+^ isolate assigned to the novel sequence type ST919. Reanalysis of its publicly available genome shows that TUM20227 belongs to cryptic clade C-III and carries a chromosomally encoded, monotoxin PaLoc-like locus encoding a 2,367-amino acid TcdB-family protein. This TcdB-like protein shares less than 83% amino acid identity with all recognized TcdB subtypes (B1–B12) and forms a deeply divergent branch in both full-length and domain-resolved phylogenies, with heterogeneous nearest neighbors across domains and an atypical predicted receptor-binding profile. TcdB_TUM20227_ should therefore be regarded as representative of a previously unrecognized, deeply divergent TcdB lineage.

**IMPORTANCE:**

*Clostridioides difficile* is a leading cause of diarrhea, and its principal virulence factor, the toxin TcdB, is both a diagnostic target and a template for therapeutics. The recognized diversity of TcdB is captured in a scheme composed of 12 subtypes largely built from strains of the five *C. difficile* clades, whereas the *Clostridioides* cryptic clades and their toxins remain comparatively underexplored, even though they can encode divergent toxins with altered receptor use and antigenicity. Here, reanalysis of a single, previously unremarked genome from a One Health survey uncovers a deeply divergent and apparently complete and intact TcdB-family toxin gene whose product falls outside the established subtype framework. This finding shows that systematic data mining of animal, human, and environmental strains can unearth novel toxin genes and provide critical insight into the evolution, receptor specificity, and subtype boundaries of the toxin genes of this important human pathogen.

## MATTERS ARISING

**Fig 1:**
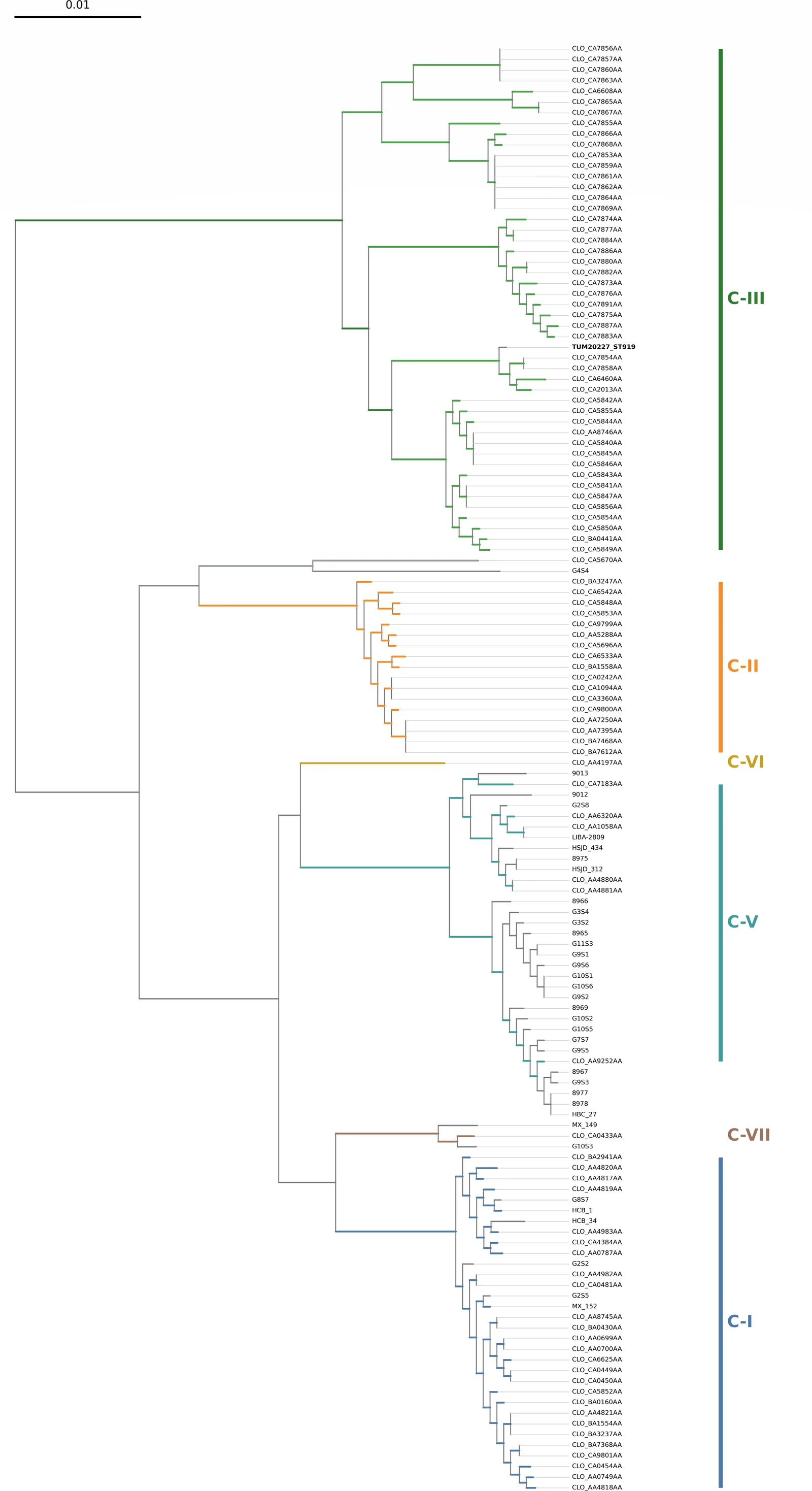
Phylogenetic placement of TUM20227 (ST919) within cryptic clade C-III. Maximum-likelihood phylogeny (FastTree) inferred from concatenated MLST allele sequences and midpoint-rooted. Strains whose identifiers begin with “CLO_” were retrieved from EnteroBase. All remaining genomes belong to the LIBA collection, within which isolates following the GxSx naming pattern are of soil origin, and the rest are of human origin. Branches are colored by cryptic clade, and clades C-I, C-II, C-III, C-V, C-VI, and C-VII are indicated; TUM20227 (in bold) is nested within clade C-III (subgroup C-IIIb). Scale bar, substitutions per site.

**Fig 2:**
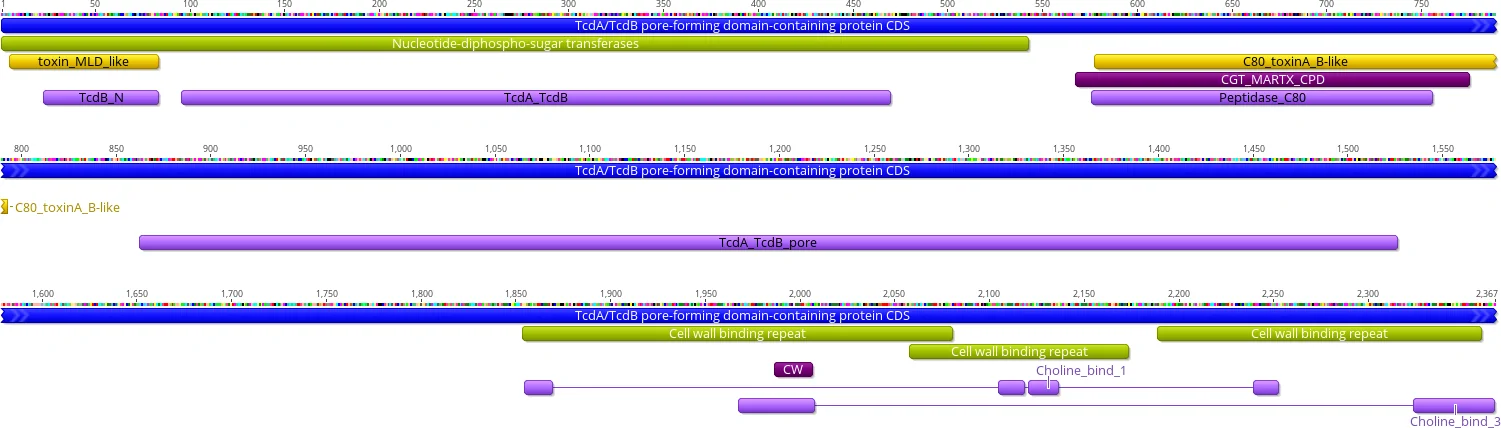
Schematic representation of sequence features detected in the TUM20227 TcdB-like toxin, confirming a typical LCT composition.

I read with great interest the article by Yoshizawa et al., “One Health genomic insights into environmental and animal reservoirs of community-associated *Clostridioides difficile* in Japan” (1). In the supplementary metadata, strain TUM20227 is listed as a Tokyo patient isolate from a 23-year-old individual, assigned to the novel ST919, and reported as *tcdA*^−^/*tcdB*^+^ and *cdtA*^−^/*cdtB*^−^. Reanalysis of its genome sequence (GCA_052078515.1 and PRJNA1302146, BioSample SAMN50463672) confirmed that TUM20227 is a cryptic clade C-III strain (Fig. 1), carrying a chromosomally encoded protein annotated by Bakta as a “TcdA/TcdB pore-forming domain-containing protein” adjacent to a “phage holin family protein,” resembling a simplified monotoxin PaLoc-like structure. BWA remapping of the original Illumina reads (SRR34877579) to this chromosomal locus showed continuous, high-depth coverage across the entire toxin coding sequence (CDS) and its flanking region (mean ~162×, with no zero-coverage positions), and the toxin gene lies well within a ~103 kb contig rather than at a contig edge. These features argue against a local assembly artifact and support the presence of a genuine CDS. The inferred toxin is a 2,367-aa protein that retains the architecture of large clostridial toxins (LCTs), including an N-terminal glycosyltransferase region, CPD/autoprotease region, delivery/translocation, and receptor-binding region, and C-terminal repeat-rich region (2) (Fig. 2). Thus, it appears to be a complete TcdB-family protein.

**Fig 3:**
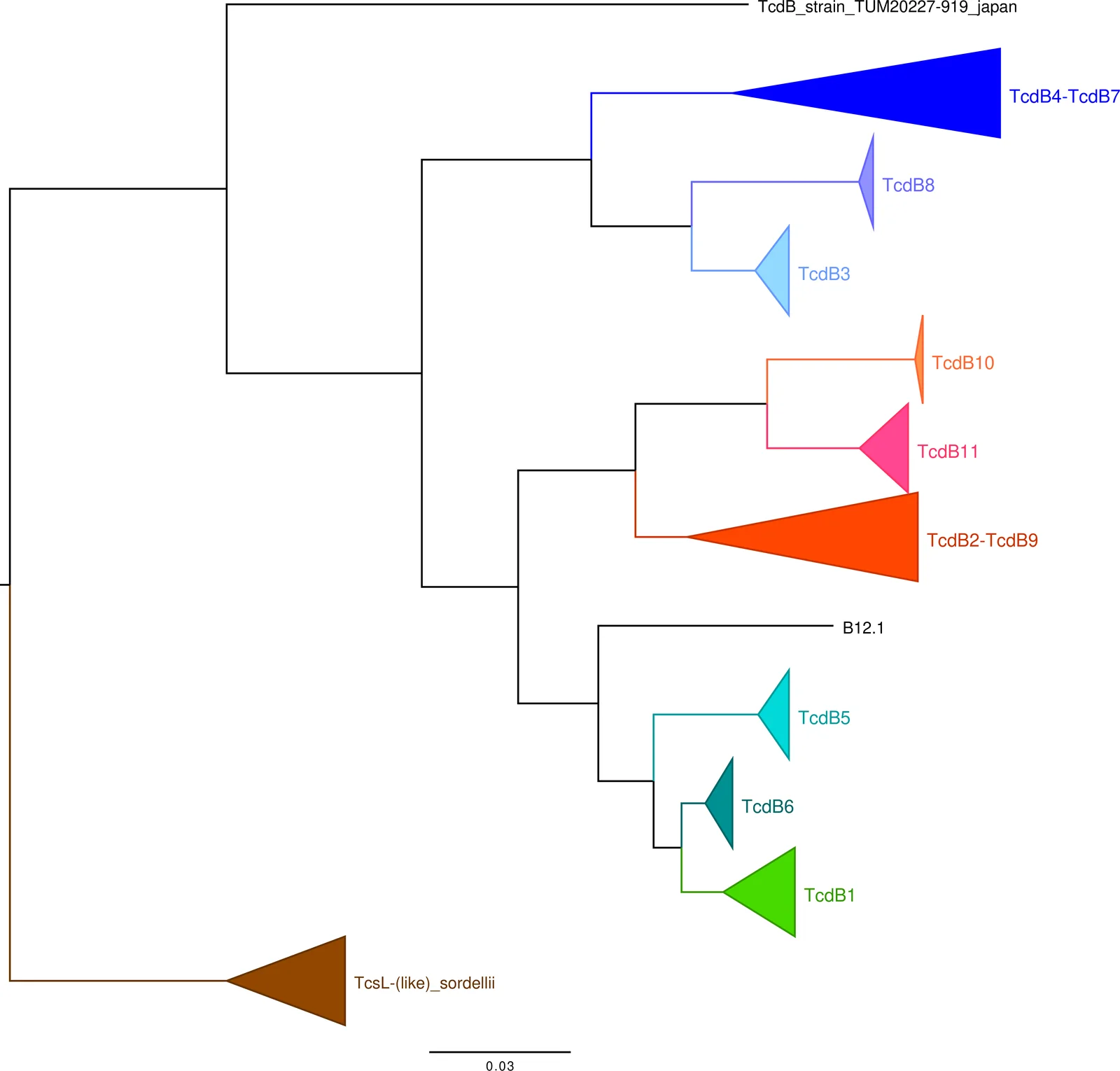
Phylogenetic placement of the TUM20227 TcdB-like toxin. Full-length TcdB/TcsL-like phylogeny showing that TcdB_TUM20227_ does not cluster within recognized TcdB subtypes B1–B12 but instead forms a distinct divergent branch.

**Fig 4:**
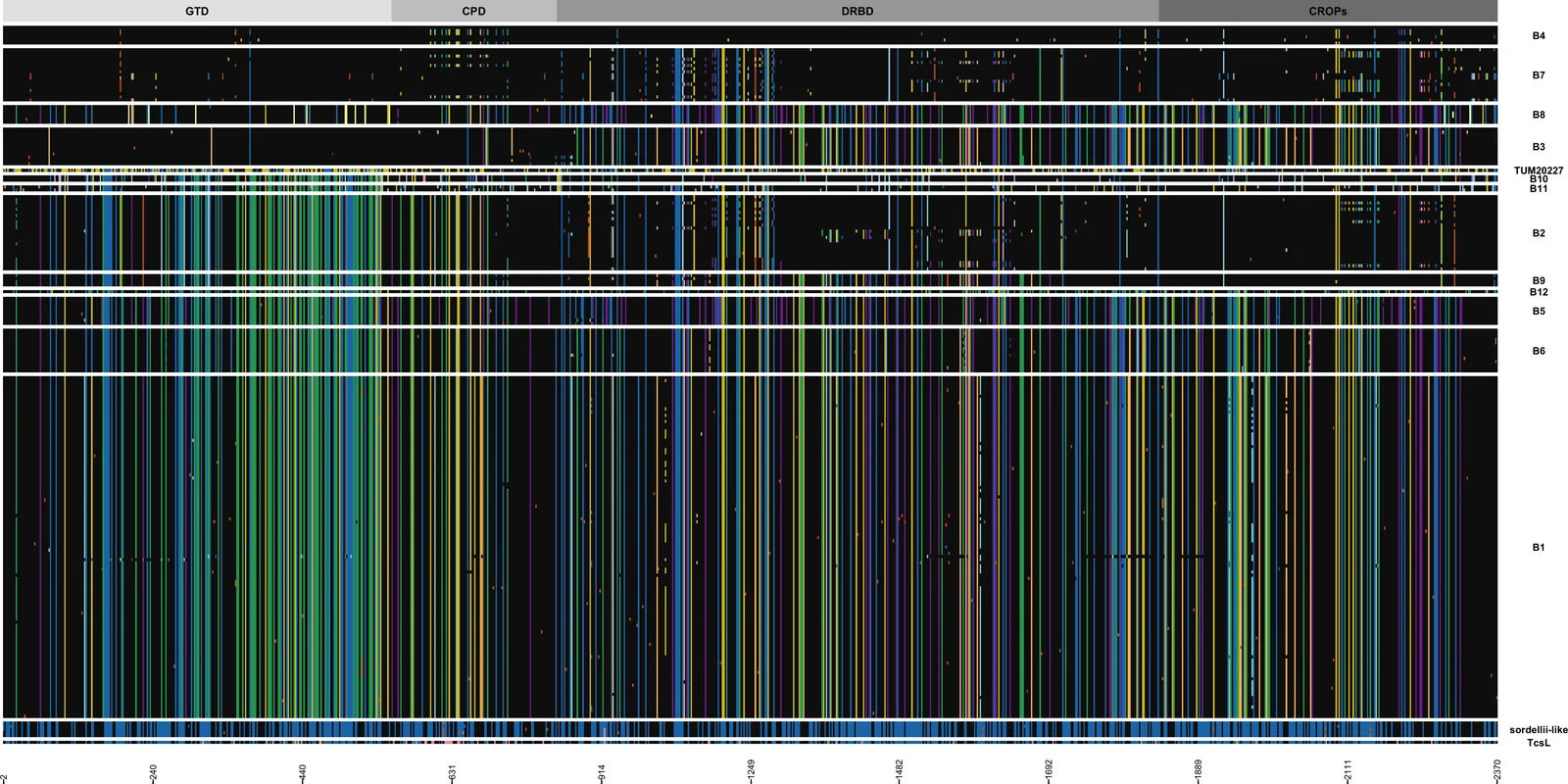
Variable-site haplotype visualization across the TcdB protein prepared with haploColor (3), with boundaries indicated for the GTD, CPD, DRBD, and CROPs domains. TcdB_TUM20227_ shows a distinct pattern relative to recognized subtypes, with its divergence distributed across the toxin rather than limited to a single sequence segment.

To assess its relationship with recognized TcdB diversity, the TUM20227 sequence was compared with full-length B1–B12 TcdB sequences from DiffBase (3, accessed 3 June). TcdB_TUM20227_ showed <83% aa identity to known TcdB proteins, far below the 97% subtype-assignment threshold (3). In a full-length ML phylogeny, TcdB_TUM20227_ did not cluster with any B1–B12 subtype but formed a deeply divergent tip (Fig. 3). Furthermore, TcdB_TUM20227_ showed a unique pattern across all GTD, CPD, DRBD, and CROPs domains in a variable-site visualization (Fig. 4).

Domain-resolved IQ-TREE analyses of the GTD, CPD, DRBD, CROPs, FZD/TFPI interface, and CSPG4-associated regions also recovered TcdB_TUM20227_ as a long-branched singleton (Table 1). Robinson–Foulds (RF) comparisons showed substantial discordance among the domain trees, with normalized RF distances of 0.910–0.976 (Table 1). Moreover, TcdB_TUM20227_ had different nearest neighbors across domains: TcsL-like/sordellii-group sequences in the GTD, TcdB4 in CPD, TcdB1 in DRBD, TcdB7 in CROPs, TcdB2 in the FZD/TFPI interface region, and TcdB11 in the CSPG4-associated region (Table 1) but did not resolve into a simple block-like mosaic of known subtype-associated domains. Because the cytopathic phenotype of LCTs is dictated largely by the GTD, a variant, TcsL-type cell-rounding morphology arising from modification of the Ras subfamily rather than RhoA GTPases would be predicted for TcdB_TUM20227_ if the protein is expressed and active, based on the TcsL/sordellii-like affinity of its GTD (Table 1) and its TcdB-range length. This prediction remains to be tested experimentally.

**TABLE 1:** Domain-resolved phylogenetic behavior of the TUM20227 TcdB-like toxin

Region	Residues	Aligned sites	Parsimony- informative sites	TUM20227 terminal branch	Nearest group	Nearest patristic distance
GTD	1–543	540	200	0.286	TcsL-like/sordellii group	0.416
CPD	544–829	286	84	0.108	B4	0.119
DRBD	830–1833	1,003	325	0.106	B1	0.190
CROPs	1832–end	536	187	0.079	B7	0.169
FZD/TFPI interface	1430–1605	176	89	0.173	B2	0.277
CSPG4-associated region	1750–1850	101	49	0.189	B11	0.383

Receptor-associated regions interpreted using published FZD-, TFPI-, and CSPG4-associated residue frameworks (3–6) suggest an atypical receptor-binding mechanism for TcdB_TUM20227_. This protein preserves 12/17 FZD-contact residues but differs from TcdB1.1 at five positions and does not match the B4/B7-like or TcdB10-like TFPI-associated receptor-switching configurations. In contrast, the CSPG4-associated interface is more conserved, with only 4/21 differences from B1.1 and retention of D1812, making CSPG4 involvement plausible but unproven.

Constrained IQ-TREE analyses forcing TcdB_TUM20227_ into each sufficiently represented subtype group (B1–B9), followed by AU, SH, and ELW topology tests, supported heterogeneous phylogenetic signal or unsampled mosaic ancestry for TcdB_TUM20227_.

Notably, TUM20227 was recovered from a patient with diarrhea and yielded a positive toxigenic culture in the original study (1). Together with the apparently intact, full-length toxin gene analyzed here, this is consistent with TcdB_TUM20227_ being a functional toxin rather than a nonfunctional relic. However, because toxigenic culture does not attribute activity to a specific protein, direct functional characterization will be required to establish the biological activity of this protein.

In conclusion, TcdB_TUM20227_ should be considered representative of a previously unrecognized, deeply divergent TcdB lineage pending formal subtype classification and functional characterization. This observation does not challenge the work of Yoshizawa et al. Instead, it underscores the value of their One Health framework and opens avenues for research on PaLoc diversification, TcdB subtype boundaries, receptor use, and functional consequences of deep toxin divergence.

## EDITOR'S NOTE

**Ed. Note**: The corresponding author of the original article read the Matters Arising, did not object to its publication, and did not feel a response was warranted.
